# Effect and Mechanism of Metakaolin Powder (MP) on Rheological and Mechanical Properties of Cementitious Suspension

**DOI:** 10.3390/ma15165797

**Published:** 2022-08-22

**Authors:** Hengrui Liu, Zezhu Wang, Zhenghong Tian, Jingwu Bu, Jianchun Qiu

**Affiliations:** 1 College of Water Conservancy and Hydropower Engineering, Hohai University, Xikang Road No. 1, Nanjing 210098, China; 2 PowerChina Guiyang Engineering Corporation Limited, Guiyang 550081, China; 3 College of Hydraulic Science and Engineering, Yangzhou University, Yangzhou 225009, China

**Keywords:** metakaolin powder, flowability and rheology, mechanical properties, water film thickness, bonding strength coefficient

## Abstract

The effects of metakaolin powder (MP) on the microscopic rheological properties and macroscopic flow parameters of cementitious suspension under various water–cement ratios were investigated. By analyzing the changes in the bonding strength coefficient and water film thickness (WFT), the mechanism of MP on flow and rheological parameters can be explored. Further, the effect of MP on mechanical properties was explained from the perspective of water absorption kinetics and hydration activity contribution rate. The incorporation of MP can reduce the flow rate and flow spread and increase the compressive strength, plastic viscosity, yield stress and thixotropy, and the effects of MP were distinctive under various W/CM ratios. The bonding strength coefficient and WFT increased and decreased with increasing MP replacement content, respectively. The regression analysis results revealed that the bonding strength coefficient and WFT were the most important factors influencing the macroscopic flow parameters and rheological parameters, which indicated that MP influenced the rheology and flowability of cementitious suspension by affecting the flocculent structure and particle distance. Compared with WFT, the bonding strength coefficient had a stronger effect on these parameters. The MP improved the compressive strength by reducing the average pore size and porosity and increasing the pore uniformity and hydration activity contribution rate of hardened paste, and this improvement was enhanced by increasing curing age.

## 1. Introduction

Metakaolin is an anhydrous aluminum silicate formed by calcining kaolin at a temperature of 600–900 °C. Due to high-temperature calcination, the layered structure of the kaolinite crystal phase is destroyed, showing a disordered molecular structure. The silicon and aluminum atoms are converted into active substances, and the external manifestation is that the metakaolin has pozzolanic activity. Under the action of an alkaline activator, metakaolin can produce calcium silicate hydrate and calcium aluminate hydrate in cement-based materials, thereby having gel properties to enhance the strength and compactness of cement-based materials [1,2,3]. Metakaolin has been widely used in geopolymers, paints and coatings, paper, rubber, wire and cable, ceramics, refractories, petrochemicals, concrete mortar and high-strength and high-performance concrete [4,5,6,7].

Green mineral admixtures include natural pozzolans, industrial waste ash and slag and other building materials, such as fly ash, zeolite powder, basalt powder, and silica fume, etc. It has the characteristics of no pollution to the environment, recycling of waste, and low price. In addition, the incorporation of mineral admixtures can replace cement to reduce the amount of cement and reduce the heat of hydration, improve the internal structure of cement-based materials through pozzolanic reaction, and thus have the characteristics of enhancing durability and, subsequently, mechanical properties. In recent years, as a new type of green mineral admixture, metakaolin has been attracting more and more interest from researchers of cement-based materials. The atoms in metakaolin are irregularly arranged, exhibiting a thermodynamically metastable state and high pozzolanic activity. Metakaolin can react with cement hydration product Ca(OH)_2_ to form gelling substances such as calcium aluminate hydrate and calcium silicate hydrate (CSH) gel, so it has high hydration activity [8,9]. Zhaohui Qin found that the replacement of metakaolin reduced the workability of the mortar but improved the compressive strength, tensile bond strength and water resistance, and the mortar mixed with 30% metakaolin had the best properties except for flowability [10]. Diandian Zhao reported that cementitious suspension samples incorporating 40% low-quality metakaolin could still achieve compressive strength comparable to that of the reference sample, and he found that Al/Ca increased with the increase in metakaolin by microscopic testing, while Ca/Si was less affected by metakaolin [11]. Ali Mardani-Aghabaglou compared the effect of various mineral admixtures on the mechanical properties and durability of mortar, such as dynamic elastic modulus, compressive strength, transmission properties, ultrasonic pulse velocity, freeze-thaw resistance and sulfate resistance. The order of the excellent performance of each mineral admixture is silica fume > metakaolin > fly ash > control sample [12]. C.-S. Poon found that the pozzolanic reaction rate and CH consumption rate of cementitious suspensions containing metakaolin were higher than those of SF or FA-containing suspensions. The high pozzolanic activity of metakaolin can effectively improve the strength development and pore structure refinement rate of early cementitious suspension. The combined use of metakaolin and other mineral admixtures in the field of cement-based materials has also been studied [13]. Jianqiang Wei believed that the combined addition of two bentonites and metakaolin could lead to an increase in the solubility of metakaolin, which reduces heat release and enhances the hydration strength of cement. The increased consumption of silicate in the cementitious suspension increased the formation of a long-chain calcium silicate hydrate (CSH) phase [14]. M. Antoni reported that 30% metakaolin and 15% limestone replacement cement had better mechanical properties than the benchmark cementitious suspensions. Calcium carbonate reacted with alumina in metakaolin to form an auxiliary AFm phase and stabilize ettringite, thereby increasing the compactness of the internal structure of the cementitious suspension [15]. Similarly, Jin Tang also found that the mixing of metakaolin and limestone brings good results. When the mass ratio of the two was as high as 1:2, the strength of the mortar was higher than that of Portland cement. The synergistic effect of metakaolin and limestone improved pore distribution by consuming silicate during hydration [16].

The above discussion showed that metakaolin had a lot of research on the mechanical, durability, and microscopic properties of cement-based materials. However, there were still few studies on the flowability and rheology of cement-based materials containing metakaolin, and there was a lack of in-depth research on the mechanism of metakaolin on the fresh properties. A few researchers have studied the macroscopic flow properties of metakaolin. Aiswarya found that when the content of NMK was 20%, the standard consistency water demand of cementitious suspension increased by 36.6% [17]. Similar studies also showed that when the NMK content was increased from 0% to 6%, the flowability of mortar was lower than the benchmark, increasing from 5.61% to 12.47% [18]. The influence mechanism of metakaolin on the macro-flowability and micro-rheology of cementitious suspension was still unclear, and this mechanism needed to be further investigated. This research was particularly important for the application of metakaolin in some engineering fields, such as in the field of 3D printing, the field of cementing, the preparation of self-compacting concrete, etc.

The research significance of this paper was as follows:

(i) The effects of metakaolin powder (MP) on the macroscopic flow parameters (flow rate and flow spread), microscopic rheological parameters (thixotropy, plastic viscosity, yield stress) and mechanical properties of cementitious suspension under various water–cement ratios were studied, which provided guidance for the application of MP in engineering.
(ii) The bonding strength coefficients and water film thickness were calculated to explain the changes in macroscopic flowability and microrheological properties, which analyzed the mechanism of MP on flow rate, flow spread, thixotropy, plastic viscosity, yield stress from the perspective of particle distance and flocculent structure and built a new evaluation system.
(iii) The improvement of mechanical properties by MP was explained from the perspective of water absorption kinetics and the hydration activity contribution rate.

## 2. Materials and Methods

### 2.1. Materials 

In this paper, ordinary Portland cement with a strength grade of 42.5 was used, which conformed to Chinese Standard GB175-2007. Metakaolin powder (MP) was an ultrafine powder formed by dehydration and ultrafine grinding at an appropriate temperature (600–900 °C). The solid densities of MP and OPC were 2200 and 3149 kg/m^3^, respectively, and were calculated according to BS EN 1097:Part 3: 1998, t.

**Figure 1:**
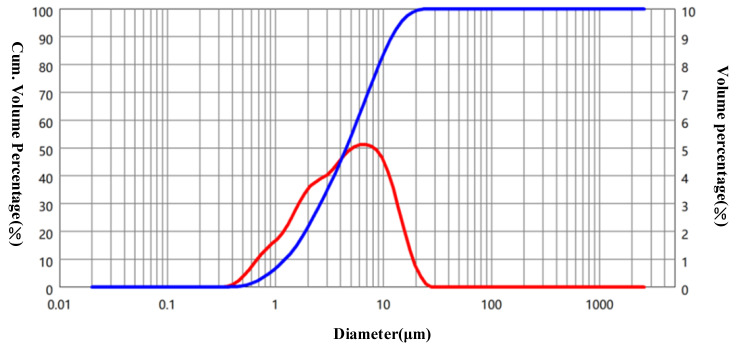
Particle size distributions of MP.

**Figure 2:**
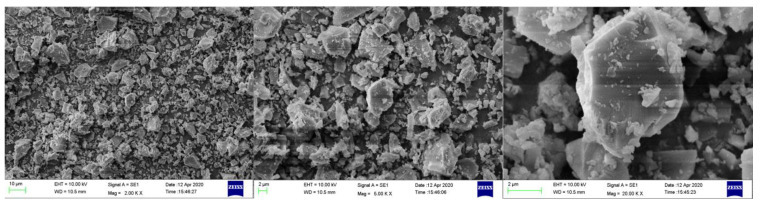
SEM morphology of MP.

Figure 1 shows the particle size distributions of MP and OPC. The median particle diameters of MP and OPC were 4.5 and 14.5 µm, respectively, and the specific surface areas of MP and OPC were calculated as 789.4 and 367.9 m^2^/g. Table 1 presents the X-ray fluorescence (XRF) results of the MP and OPC. The total contents of Fe_3_O_4_, Al_2_O_3_ and SiO_2_ in the MP were 90.22%, which conformed to ASTM C 618. The particle shapes and morphologies of the MP are shown in Figure 2. The MP had good particle gradation and irregular particle shape, so MP had no lubricating effect. The MP mainly affected the microscopic rheology and macroscopic flowability of cementitious suspension through the filling effect and surface effect.

### 2.2. Experimental Program

This research plan was divided into three parts. In the first part, the rheology, flowability and mechanical properties of the cementitious suspension containing MP were tested under various W/CM ratios to observe the effect of MP on the fresh properties. The water–cement ratio ranged from 0.5 to 0.7 and increased in steps of 0.5, and the MP replacement contents were 5%, 10%, 15%, and 20%, respectively. In the second part, the void ratio and packing density of the suspensions were tested to calculate the water film thickness (defined as WFT), and the flocculated structure’s strength and size of the cementitious suspension were tested to calculate the bonding strength coefficient. The variations of WFT and the bonding strength coefficient can reflect the influence of MP on micro-rheological parameters and macro-flow parameters. Finally, the mechanism of MP on the compressive strength of hardened cement paste was studied from the water absorption kinetics and the hydration activity contribution rate. The cementitious suspension samples were produced in a controlled environment at a 95% humidity and temperature of 20 ± 2 °C. 

### 2.3. Test Methods

#### 2.3.1. Flow Spread

The flow spread of cementitious suspension was tested using mini-slump cones, and the micro-slump cones used were the same as those used by Okamura and Ouchi [19].

The procedure for the mini slump test was as follows:

(i) The slump cone was placed in the center of the flat steel plate.
(ii) The cementitious suspension was slowly poured into the slump cone until the slump cone was completely filled;
(iii) The slump cone was lifted to allow the suspension to spread until it stopped.
(iv) The diameter of the suspension cake was measured in two orthogonal directions, and the average diameter was calculated. The flow spread of the cementitious suspension was the mean diameter that reduced the bottom diameter of the slump cone.

#### 2.3.2. Flow Rate

The marsh cone model with dimensions of 94 × 30 × 7 mm was adopted to test the flow rate according to JC/T 1083-2008, and the initial Marshall time was recorded to calculate the flow rate [20]. The test procedure was described below, the marsh cone was mounted on the stand, and the graduated cylinder was placed under the orifice of the marsh cone. The orifice was closed, and 300 mL of cementitious suspension was poured into the marsh cone. The time required for 300 mL of suspension to flow out was measured. The flow rate was determined as 300 mL divided by the measurement time.

#### 2.3.3. Plastic Viscosity, Yield Stress and Thixotropy

The plastic viscosity, yield stresses and thixotropy of different cementitious suspension samples were tested by an Rvdv1-T rotary viscometer [21,22]. The shear stress at different shear rates was tested, and regression fit analysis was performed on the test data points. Shear stress and shear rate followed a certain mathematical relationship. The most widely used in the field of cement-based materials were the Bingham model, the improved Bingham model, and the Herschel–Bulkley Model [23]. The improved Bingham model in this paper was considered to be the most suitable rheological model for cementitious suspension containing MP. 

The rheological parameters can be calculated from the following equation:

(1) $$\tau =\tau_{0}+\mu \times \gamma +c\times \gamma^{2}$$

where *γ* is the shear rate; *μ* is the plastic viscosity, and c is the second-order parameter; *τ*_0_ and *τ* are yield stress and shear stress. 

Thixotropy was measured as follows: By continuously increasing the shear rate of the rotor, an upward shear rate-shear stress curve was obtained. At this time, due to the continuous increase in the rotational speed, the speed of particle structure reformation always lagged behind the destruction speed of the structure, and the matter system was in a non-equilibrium state. When a certain maximum rotational speed was reached, the rotational speed was gradually reduced. Since the damaged structure cannot be reformed in time, the shear stress was only used to make the material flow, and the downward shear rate–shear stress curve was obtained. The area between the upward and downward curves was defined as thixotropy.

#### 2.3.4. Compressive Strength

The specific steps of the compressive strength test in this experiment were carried out according to the Chinese standard GB50204-2002. The compressive strength test block of the cement paste was a cube, and the size of the test block was 70.7 × 70.7 × 70.7 mm. The poured test block was put into a curing room with a humidity of 95 °C and a temperature of about 20° for 7 and 28 days of curing.

#### 2.3.5. Water Film Thickness (WFT)

The void ratio and packing density needed to be tested to calculate the WFT. The wet packing test method proposed by Kwan was used to measure the packing density of each cementitious material mixture [24,25]. The maximum solids concentration was achieved when the cementitious material was mixed with water at various water–cement ratios, and packing density was defined as the maximum solids concentration of a mixture. To measure packing density, starting from a relatively high W/CM ratio, the W/CM ratio was successively decreased until the solids concentration reached a maximum value and then decreased, resulting in six to eight grout samples of the cementitious material. The *WFT* can be calculated by void ratio and packing density, and the formulas were as follows:

(2) $$u=\frac{\left(1-\Phi_{\max}\right)}{\Phi_{\max}}$$

(3) $${u\prime }_{w}=u_{w}-u$$

(4) $$A=A_{0}\times R_{0}+A_{M}\times R_{M}$$

(5) $$WFT=\frac{{u\prime }_{w}}{A}$$

where ${u\prime }_{w}$ is the free water available for suspension flow; $\Phi_{\max}$ is the packing density; u is the void ratio; $u_{w}$ is the volume of the mixing water; *R*_0_ and *R*_M_, are the volumetric ratios of the OPC and MP to the total solid volume, respectively; *A*_0_, and *A*_P_ are the specific surface areas of OPC and MP, respectively.

#### 2.3.6. Bonding Strength Coefficient

The flocculent structure of cementitious suspension was tested by a multi-screen separation method [22,26]. Cementitious suspension was a heterogeneous medium composed of different size and strength flocculent structures. The principle of the sieve mesh screening flocculent structure of suspension was that the large size of the flocculent structure could not pass through the low aperture of the sieve, while the flocculent structure smaller than the aperture of the screen directly passed through. In this study, the flocculent structure of cementitious suspension was classified by five apertures. Sieves with apertures of 150, 300, 425, 600, 830 μm and 30 cm in diameter were used, and the weight of the sieve was measured and recorded as m_1_, m_2_, m_3_, m_4_, and m_5_, respectively. As an example of the 150 μm sieve test, the sieve was positioned in a cylindrical mold 10 cm high and 28 cm in diameter, and both were positioned on the scale and the scale was zeroed. The cementitious suspension was poured evenly and slowly into the sieve. As part of the cementitious suspension stayed in the beaker, the total mass selected here was 260 g. When the poured suspension was no longer dripping on the screen, the suspension and sieve were moved to a scale for weighing, with a mass of m_1_′. Then, we repeated the above test steps on the sieve of 300, 425, 600 and 830 μm, with the mass of m_2_′, m_3_′, m_4_′ and m_5_′, respectively. Formula (6) was used to calculate the mass fraction of cementitious suspensions with different sizes of flocculent structures:

(6) $$\begin{array}{l} M_{>150\mathrm{um}}=\frac{m_{1}\prime -m_{1}}{260}\;M_{<150\mathrm{um}}=1-\frac{(m_{1}\prime -m_{1})}{260}\\ M_{>300\mathrm{um}}=\frac{m_{2}\prime -m_{2}}{260}\;M_{150–300\mathrm{um}}=\frac{(m_{2}\prime -m_{2})-(m_{1}\prime -m_{1})}{260}\\ M_{>425\mathrm{um}}=\frac{m_{3}\prime -m_{3}}{260}\;M_{300–425\mathrm{um}}=\frac{(m_{3}\prime -m_{3})-(m_{2}\prime -m_{2})}{260}\\ M_{>600\mathrm{um}}=\frac{m_{4}\prime -m_{4}}{260}\;M_{425-600\mathrm{um}}=\frac{(m_{4}\prime -m_{4})-(m_{3}\prime -m_{3})}{260}\\ M_{>830\mathrm{um}}=\frac{m_{5}\prime -m_{5}}{260}\;M_{600–830\mathrm{um}}=\frac{(m_{5}\prime -m_{5})-(m_{4}\prime -m_{4})}{260} \end{array}$$

When the mass fraction of the flocculent structure of different sizes of the cementitious suspension was obtained, the area method could be used to quantify flocculent structure’s size to obtain the size parameters of the flocculent structure, and the parameter was recorded as *a*. The fractal dimension can reflect the flocculent structure’s strength of cementitious suspension, which was related to the size, density, void ratio of the flocculent structure and the properties of the constituent particles [27,28]. The formula for calculating the fractal dimension was as follows [29]:

(7) $$D_{f=}\frac{3+3\mathrm{In}\;(1-\mu )}{\mathrm{In}\;D/d-\mathrm{In}\;(1-\mu )}$$

where *D*_f_ is the fractal dimension; u is the void ratio; D is the flocculent structure’s size; d is the size of the composed particle.

The fractal dimension can be further analyzed by mathematical methods to obtain the strength parameters that characterize the flocculent structure, and the parameter was recorded as *b*. The detailed calculation of the flocculent structure size characterization parameter *a* and the strength standard parameter *b* can be found in reference [26].

The formula for calculating the bonding strength coefficient was as follows:

(8) ζ=ab

#### 2.3.7. Water Absorption Dynamics

The water absorption kinetics method was a test method based on the capillary phenomenon for the determination of the pore structure of cement-based materials. The water absorption method can measure the integral parameter of the apparent porosity of the pore structure of the material, as well as the differential parameters such as the average pore size and pore size uniformity. It was a non-destructive testing method with the advantages of simple testing equipment and easy operation. 

The principle of the water absorption kinetics method: the water absorption kinetics of cement-based materials were closely related to the pore structure index, and the index can be obtained by establishing a cylindrical capillary model. Under isothermal conditions, when the capillary adsorption of cement-based materials occurred, the water absorption curve had a stable exponential function. The water absorption kinetics method was used to analyze their pore structure, which can be expressed as the following formula:

(9) $$W_{t}=W_{\max}(1-e^{-\lambda t^{\alpha }})$$

where *W*_t_ is the mass water absorption rate after time *t*; *W*_max_ is the maximum mass water absorption; *λ* is the average pore size of the capillary; *α* is the uniformity of the capillary pores.

The formula for calculating *W*_t_ is as follows:

(10) $$W_{t}=\frac{m_{t}-m_{0}}{m_{0}}$$

where m_0_ is the mass of the specimen after drying; m_t_ is the mass of the specimen after soaking in water for t hours; *W*_t_ is the mass water absorption rate after time *t*.

From the above, *λ* can be calculated as follows:

(11) $$\lambda =\frac{\mathrm{Ln}(1-\frac{W_{t}}{W_{\max}})}{t^{\alpha }}$$

When *t* takes 1, Formula (12) is obtained:

(12) $$\lambda =\mathrm{Ln}(1-\frac{W_{t}}{W_{\max}})$$

where *W*_t_ is the mass water absorption of the specimen after immersing in water for 1 h. According to Formula (11), the average pore diameter *λ* of the time can be calculated. Then, *λ* is substituted into Formula (9) and takes *t* = 0.25 to obtain the pore uniformity *α* of the specimen.

The parameters *λ* and *α* of the differential porosity and the parameter of the integral porosity *W*_max_ (referred to as W) can be used as indicators to evaluate the pore structure of the mortar and paste specimens. By studying the average pore size, pore uniformity and mass water absorption of the specimens, the variation law of the pore structure of the specimens with the amount of MP substitute content and the curing age was discussed.

The specific operation is as follows: after curing, the samples were tested for 7 and 28 d under the standard curing conditions, the specimens were dried, and then the quality of the specimens immersed in water for 0, 0.25, 1, and 24 h was measured and recorded as m_0_, m_0.25_, m_1_, m_24_. Finally, the mass water absorption rate W, pore average α and pore uniformity λ of the specimen were calculated according to the formula.

## 3. Results and Discussion

### 3.1. Experimental Result

#### 3.1.1. Flow Parameters

**Figure 3:**
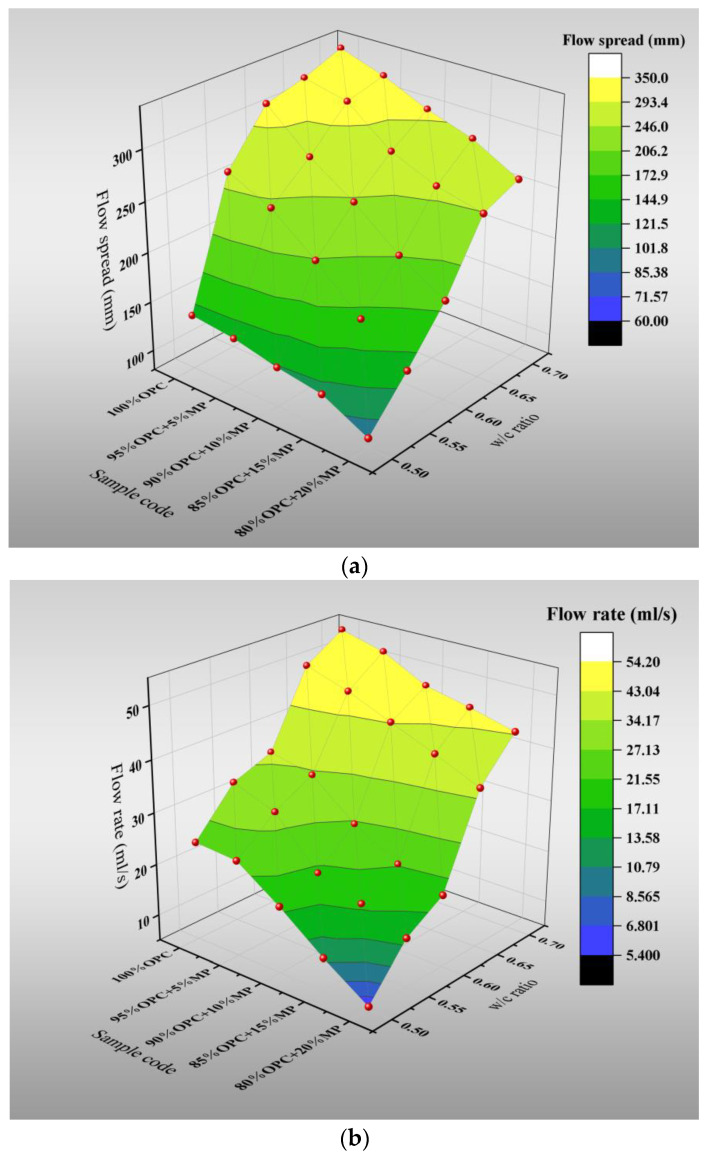
Differentiation of flow rate and flow spread with W/CM at various MP contents. (**a**) Flow spread. (**b**) Flow rate.

Figure 3 and Table 2 show the variations of flow rate and flow spread with the W/CM ratio at various MP contents. The results showed that the addition of MP significantly reduced the macroscopic flowability of the cementitious suspension. The results show that the addition of MP significantly reduces the macro-flowability of cementitious suspension. With the increase in MP content from 5% to 20%, the flow spread varied in the range of 2.4–48.8% at different W/CM ratios, and the flow rate varied in the range of 2.1–76.7% at different W/CM ratios. When the W/CM ratio was 0.7, the effect of MP on macroscopic flowability of suspension decreased to the lowest, and the flow rate and flow spread decreased by 4.08%, 12.2%, 15.7%, 19.7% and 4.47%, 10.44%, 14.92%, 22.38%, respectively.

The addition of MP weakened the macroscopic flowability of the cementitious suspension because of the high specific surface area of MP, which required more free water to wrap around the particle surface to form a water film. The reduction in the WFT increased the friction and collision between particles and weakened the flowability of the suspension [30,31]. In addition, the incorporation of MP increased the electrostatic attraction to make the inter-particle linkages tighter, which increased the flocculent structure’s strength and size and hindered the flow of the suspension.

#### 3.1.2. Rheological Parameters

Yield stress is defined as the critical shear stress required to make the suspension flow. Only when the external shear stress is greater than the yield stress can the suspension flow; otherwise, only the internal structure can be deformed. The plastic viscosity is an index to characterize the internal friction properties of the cementitious suspension. It can reflect the degree to which the internal structure hinders the flow of the suspension. Thixotropy is a reversible sol phenomenon that occurs under the action of mechanical forces such as vibration and compression. Plastic viscosity, yield stress, and thixotropy reflected the micro-rheology of the suspension and indirectly reflected the influence of the internal structure on the flowability. Rheological parameters play a vital role in some engineering fields, such as the cementing field, 3D printing, controlling SCC template pressure, etc.

**Figure 4:**
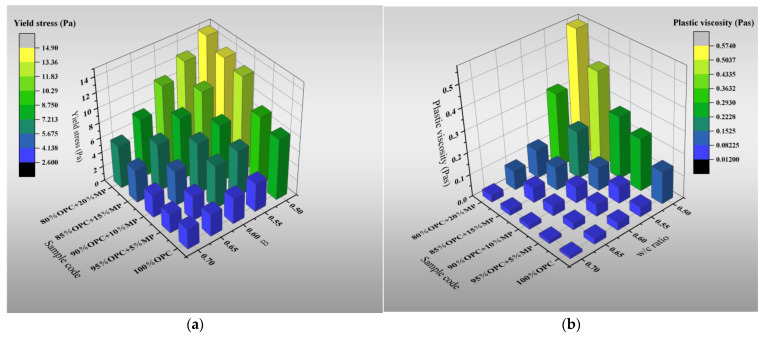

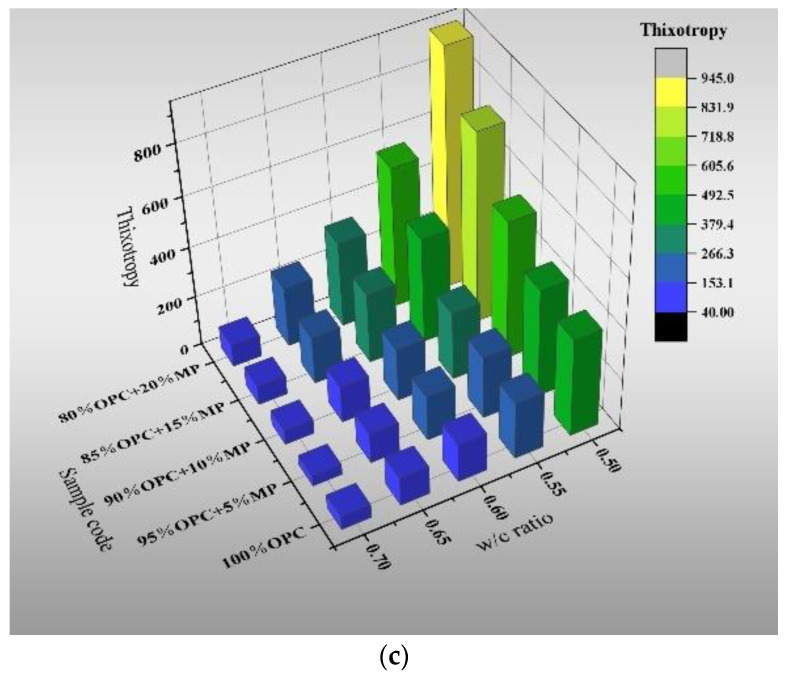
Differentiation of plastic viscosity, yield stress and thixotropy with W/CM at various MP contents. (**a**) Yield stress. (**b**) Plastic viscosity. (**c**) Thixotropy.

Figure 4 presents the variations of plastic viscosity, yield stress and thixotropy with the W/CM ratio at various MP contents. The results indicated that MP could increase the rheological parameters of cementitious suspension, but the effect was distinctive under different W/CM ratios. As the W/CM ratio increased, the yield stress varied in the range of 5.1–234.2%, with an increase in MP substitute content from 5% to 20%, the plastic viscosity varied in the range of 1.2–661.3%, and the thixotropy varied in the range of 1.8–146.69%. The changes in yield stress and plastic viscosity belong to the normal range. According to the research of Zhenghong Tian [32], with an increase in the W/CM ratio, the yield stress varied in the range of 3.6–222.3%, with an increase in ZP substitute content from 5% to 20%, and the plastic viscosity varied in the range of 0.8–489.7%. Similar to the change rule of flow parameters, as the W/CM ratio increases, the effect of MP on the rheological parameters weakens. When the W/CM ratio was 0.7, the effect of MP on the micro-rheological parameters was minimized. The yield stress increased by 5.45%, 12.7%, 63.6%, 110.9%, the plastic viscosity increased by 25%. 50%, 108%, 158%, and thixotropy increased by 1.8%, 7.8%, 41.2%, 65.4%, with the increase in MP replacement content from 5% to 20%. It is worth noting that the values of the rheological parameters of the cementitious suspension were at a low level at high W/CM ratios, especially the plastic viscosity. The plastic viscosity of all proportions did not exceed 0.1 when the W/CM ratio was larger than 0.6. The result showed that MP played a little role at high W/CM ratios, and the rheological parameters between the different sample codes were not remarkable.

Since the particle size of MP was finer than that of the cement particles, it can effectively fill the voids between the cement particles within the suspension. The increase in packing density made the strength of the internal cementitious network structure increase, which can withstand greater external stress, so the addition of MP increased the yield stress. The plastic viscosity was closely related to the flocculent structure inside the suspension, and the addition of MP increased the electrostatic attraction between particles, which increased the flocculent structure’ strength and size. In addition, the addition of MP increased the nucleation sites of cement particle hydration, and the increase in hydration products also increased the strength of the flocculent structure. Therefore, the ability of internal structures in a suspension to impede the flow increased, and the plastic viscosity increased. Thixotropy was related to the cohesion between particles, and the addition of MP enhanced the cohesion between particles. When the suspension structure was damaged by external action, the particles can quickly recombine and flocculate to form new flocculated structures.

The reason why MP played different roles in different W/CM ratios was that there was a threshold value of water film thickness. When the water film thickness exceeded this threshold, the effect of mineral admixtures on microscopic rheological parameters weakened, which was related to the microscopic forces existing between suspension particles. The microscopic forces inside the suspension include electrostatic attraction, Brownian force and gravity, among which the electrostatic attraction and Brownian force are related to the particle distance, and the water film thickness can indirectly represent the particle distance. The addition of MP changed the water film thickness and affected the rheology of cementitious suspension. At high W/CM ratios, the particle spacing was large, and the filling effect of MP was limited, so it was difficult to change the particle spacing to a large extent. The effect of MP addition on colloid attraction and Brownian force was weakened, so MP played a limited role at a high W/CM ratio [32].

#### 3.1.3. Compressive Strength

**Figure 5:**
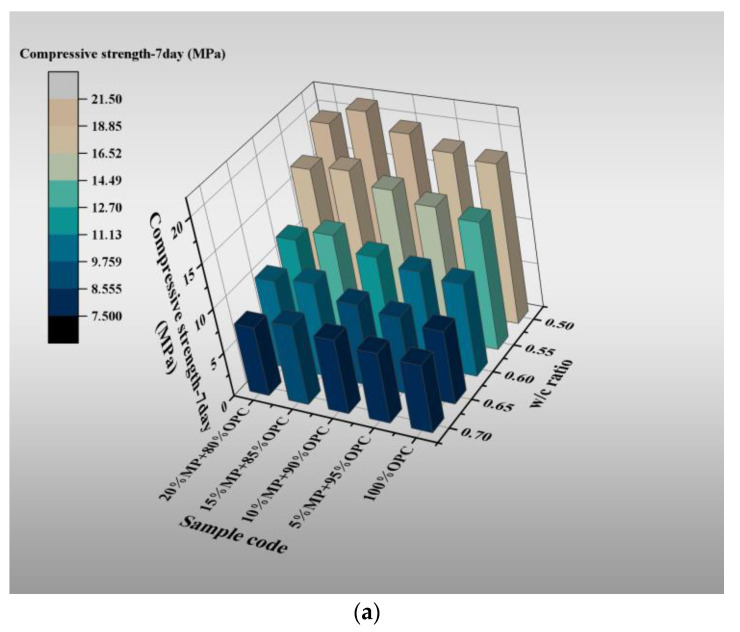

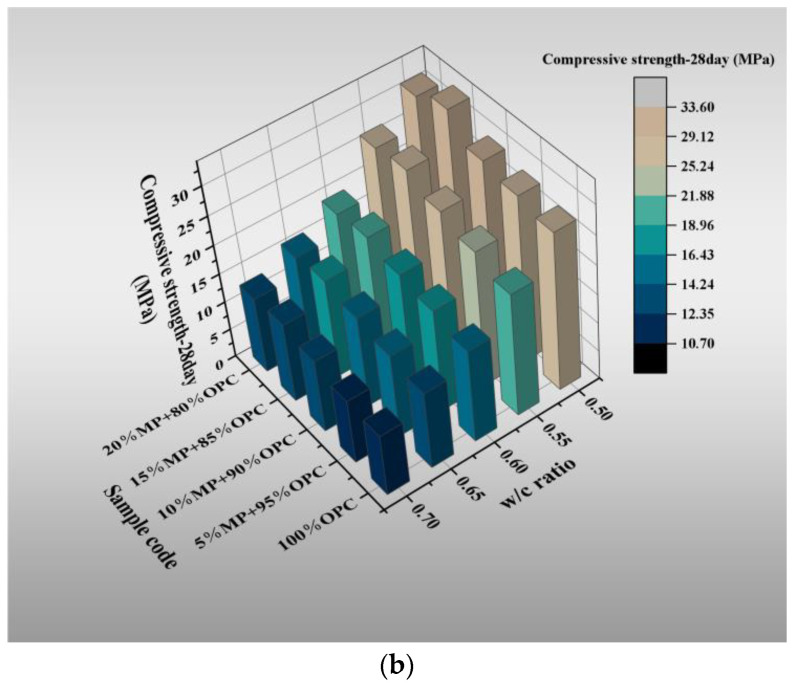
Differentiation of 7-day and 28-day compressive strength with W/CM at different MP contents; (**a**) 7 days, (**b**) 28 days.

The 7-day and 28-day compressive strength are plotted against MP contents for different W/CM ratios in Figure 5 and Table 3. The MP can effectively improve the mechanical properties of hardened slurry. The 7-day and 28-day compressive strengths of different samples had different degrees of improvement at various W/CM ratios, and they are all greater than the control. Among them, the compressive strength of the samples increased with MP substitute content from 0 to 15% and then decreased with MP substitute content from 15% to 20%, showing the same trend at various W/CM ratios. The compressive strength of 5%, 10%, 15% and 20% MP at 7 and 28 days increased by 2.8%, 10.6%, 20.7%, 10.1% and 4.4%, 8.4%, 22.7%, 15.1%, respectively, at a W/CM ratio equal to 0.5. When the W/CM ratio was 0.55, 5%, 10%, 15% and 20%, MP increased by 6.3%, 14.1%, 23.9%, 20.4% and 9.9%, 18.8%, 29.1%, 25.3%, respectively. When the W/CM ratio was 0.6, 5%, 10%, 15% and 20%, MP increased by 4.8%, 12.6%, 28.2%, 15.5% and 10.2%, 17.1%, 26.3%, 21.3%, respectively. When the W/CM ratio was 0.65, 5%, 10%, 15% and 20%, MP increased by 7.4%, 13.5%, 31.5%, 24.7% and 7.4%, 17.1%, 36.6%, 28%, respectively. When the W/CM ratio was 0.7, 5%, 10%, 15% and 20% MP increased by 4.1%, 10.6%, 18.6%, 14% and 4.3%, 18.3%, 28.5%, 22.9%, respectively. The 15% MP was the best blend for improving the mechanical properties of hardened cement paste, with the highest compressive strength at 7 and 28 days. In addition, the improvement effect of MP on mechanical properties in 28 days was better than that in 7 days, which was attributed to the volcanic ash effect of MP.

The MP can improve the mechanical properties (7-day and 28-day compressive strength) of hardened cement paste. (i) Microscopically, it was explained that MP contains high contents of active Al_2_O_3_ and SiO_2_, which can react with Ca(OH)_2_ to form hydration calcium aluminate, calcium silicate hydrate [13]. The increase in the degree of cement hydration densified the internal structure of the paste, thereby improving the compressive strength. When the replacement of MP was lower than 15%, the filling effect of MP could be exerted more effectively, and the voids inside the cement paste were partially filled with MP to further improve the compactness of the slurry. (ii) MP can induce the crystallization of cement hydration products to fill the voids and also promote the further hydration of tricalcium silicate and tricalcium aluminate, resulting in the reduction in pores in the C-S-H gel [14]. Therefore, when the MP powder content was less than or equal to 15%, the uniform distribution of MP powder in the cement paste can increase the effective crystal content and reduce the crystal defects, thereby improving the overall structural strength. When the content of MP increased to 20%, the content of cement was further reduced by the replacement of MP with high content, and the negative effect caused by the lack of cement was gradually reflected. The 20% MP was lower than 15% compressive strength but still significantly greater than the control paste. It can be expected that further attenuation of compressive strength will occur as MP replaces 20% more content. Even though MP can play a filling role and promote hydration, the beneficial effect was lower than the harmful effect caused by cement reduction. 

### 3.2. Mechanism of MP on Rheology and Flowability of Cementitious Suspension

#### 3.2.1. Water Film Thickness (WFT)

**Figure 6:**
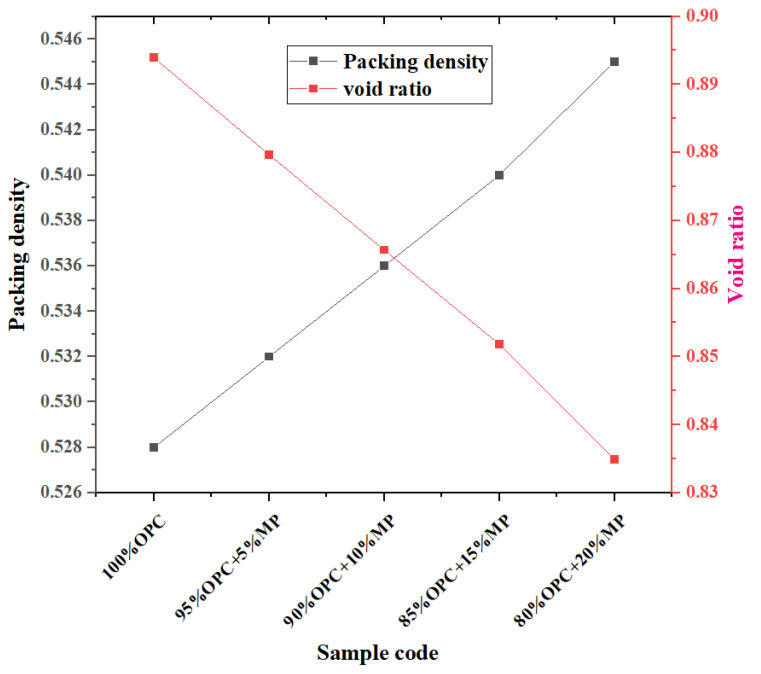
Differentiation of void ratio and packing density at different MP contents.

The void ratio and packing density are plotted against MP contents for different W/CM ratios in Figure 6. The addition of MP increased the packing density and decreased the porosity of the mixture, which was attributed to the good filling effect of the fine particle size of MP. With the addition of MP from 5% to 20%, the packing density of the mixture increased by 0.75%, 1.5%, 2.2%, 3.2%, while the void ratio decreased by 1.6%, 3.2%, 4.7%, 6.6%. Under the high substitution of MP, the void ratio of the mixture decreased, and the packing density increased obviously. The decrease in void ratio and the increase in packing density can effectively enhance the structural compaction of the cementitious suspension.

**Figure 7:**
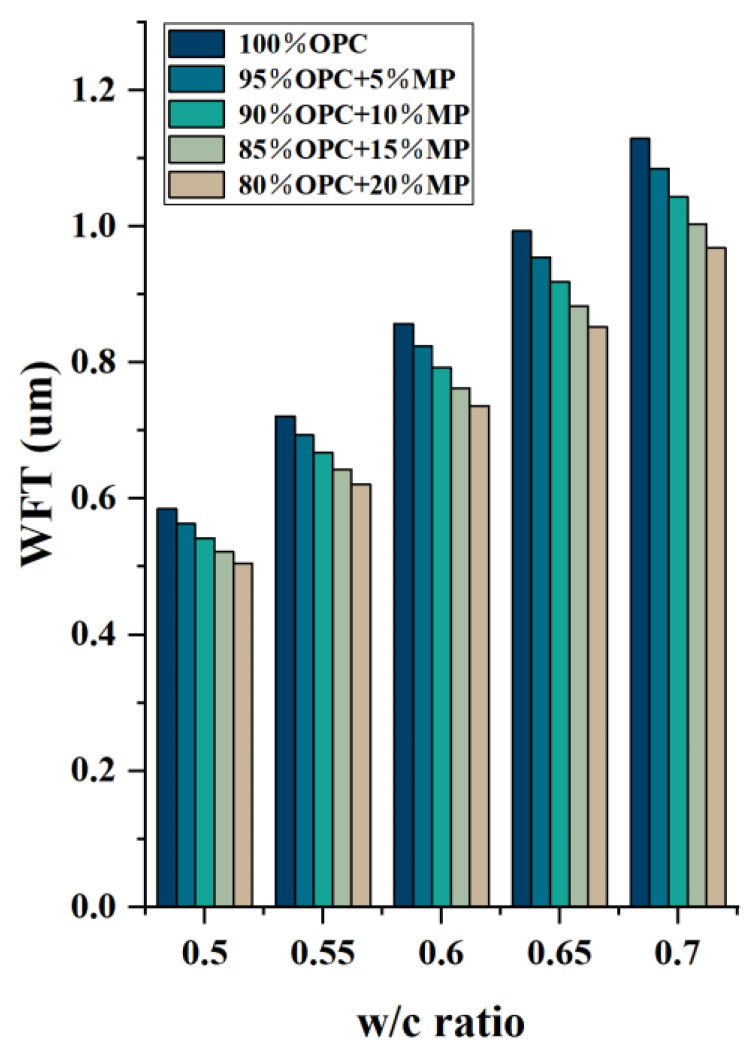
Differentiation of WFT with W/CM at different MP contents.

Figure 7 presents the differentiation of water film thickness with a W/CM ratio at different MP contents. The WFT decreased with the decrease in the W/CM ratio and the increase in substitute content of MP, which was consistent with the test results of microscopic rheology and macroscopic flowability. The WFT can be used to characterize the filling effect and surface effect of the material. The addition of MP played a filling role and released free water to enhance the flowability of suspension. However, MP also had a high specific surface area and required more free water to wrap the particle surface. The absorption of free water by the surface effect was greater than the free water released by the filling effect, so the incorporation of MP reduced the WFT within the suspension. The reduction in particle distance increased collision and friction between particles, which increased the ability of the suspension’s structure to impede its flow, the macroscopic flow parameters decreased, and the microscopic rheological parameters increased. In Section 3.2.3, the relationships between macroscopic flow parameters, microrheological parameters and WFT are evaluated.

#### 3.2.2. Bonding Strength Coefficient

**Figure 8:**
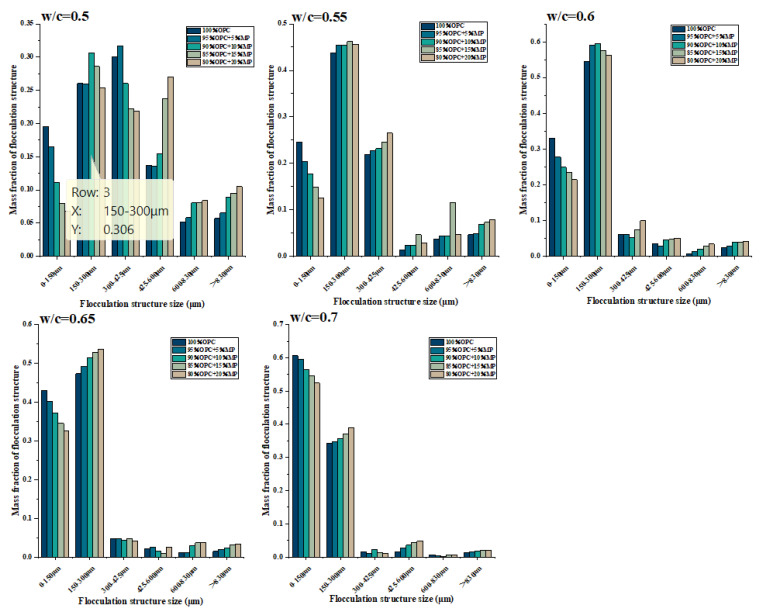
Differentiation of flocculent structure size with W/CM at different MP contents.

The mass fractions of different flocculent structure sizes are plotted against MP contents for different W/CM ratios in Figure 8 and Table 4. The W/CM ratio was the most important factor in changing the flocculent structure’s size and mass fraction. When the W/CM ratio was 0.5, the flocculent structure size range of all proportions was mainly concentrated within 0–600 μm, and the range > 600 μm accounted for no more than 10% of the mass fraction of all flocculent structures of the cementitious suspension. When the W/CM ratio was 0.55, the flocculent structure’s mass fraction in the range of 425–600 μm decreased significantly, and the flocculent structure in this range was broken and decomposed into the range of 0–300 μm. The flocculent structure in the range of 600–830 μm and >830 μm was further broken, and the mass fraction decreased. At this time, the flocculent structure was mainly concentrated in the range of 0–425 μm, and the mass fraction accounted for more than 90%. When the W/CM ratio was 0.6, the main crushing and decomposition interval of the flocculent structure was 300–425 μm, while the interval greater than 420 μm continued to be further broken, but the decrease in the mass fraction at this time was not obvious. At this time, all proportions of the flocculent structure were mainly concentrated in the 0–300 μm range. When the W/CM ratio was 0.6 and 0.65, the mass fraction of 150–300 μm was higher than that of 0–150 μm. At a W/CM ratio equal to 0.7, the flocculent structure in the range of 150–300 μm was further broken, and the mass fraction of 0–150 μm was higher than that of 150–300 μm. 

**Figure 9:**
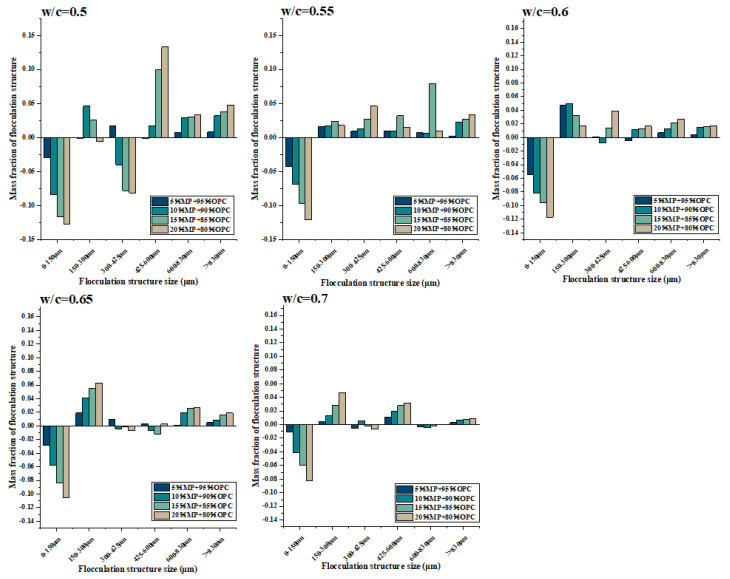
Differentiation of flocculent structure of different sizes on the basis of control cementitious suspension at different MP contents.

Figure 9 presents the variation of different flocculent structure sizes of cementitious suspension containing MP on the basis of control cementitious suspension at different water–cement ratios. The influence of MP on the flocculent structure had three common laws at various W/CM ratios: (i) The addition of MP significantly reduced the flocculent structure in the range of 0–150 μm. The more MP substitution, the more significant the decrease in the flocculent structure’s mass fraction. (ii) The flocculent structure in the range of >830 μm always showed an increasing trend, and the mass fraction did not decrease as the W/CM ratio increased. The higher the substitute content of MP, the more significant the increase in the flocculent structure’s mass fraction. (iii) The ability of MP to enhance the flocculent structure of cementitious suspension became weaker with the increase in the W/CM ratio, and the degree of increase in the large-size flocculent structure decreased with the increase in the W/CM ratio.

At a W/C ratio equal to 0.5, the addition of MP decreased the flocculent structure in the range of 0–425 μm, while the flocculent structure of 400–830 μm increased significantly. When the water–cement ratio was 0.55 and 0.6, only the flocculent structure in the range of 0–150 μm decreased, and the range of >150 μm increased in different degrees. Compared with the scenario where the W/CM ratio was equal to 0.5, the flocculent structure interval of 150–425 μm no longer decreased but increased. The flocculent structure’s mass fraction of each size was weakened to varying degrees when the W/CM ratio was further increased, indicating the influence ability of MP on the flocculent structure was weakened.

**Figure 10:**
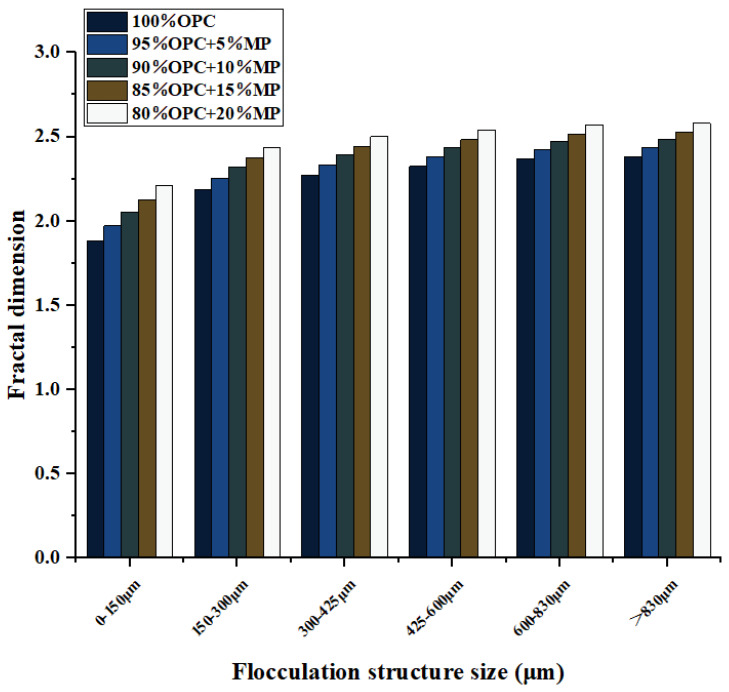
Differentiation of fractal dimension with W/CM ratios at different MP contents.

**Figure 11:**
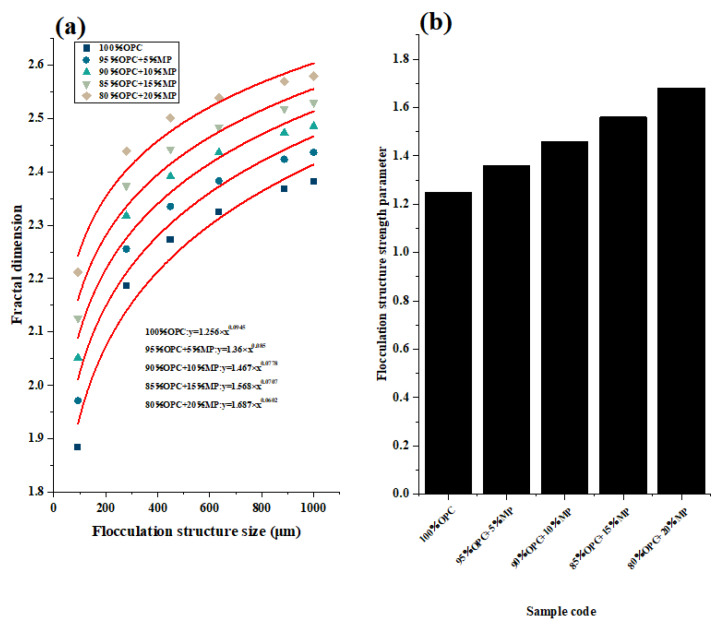
Curve fitting of fractal dimensions of different flocculation structure sizes and variations of flocculation structure strength parameter with W/CM ratios at different MP contents.

The addition of MP not only affected the flocculent structure’s size of the cementitious suspension but also changed the flocculent structure’s strength. The fractal dimension can reflect the strength of the flocculent structure. Figure 10 presents the variation of fractal dimensions of different flocculation structure sizes at different MP contents. It can be seen from Figure 11 that the addition of MP can increase the strength of the flocculent structure of different sizes within cementitious suspension, but this effect weakened with the increase in flocculent structure size and enhanced with the increase in MP content. The 5% MP was enhanced by 4.59%, 3.13%, 2.75%, 2.53%, 2.35%, 2.29% on the control suspension in the range of <150 to >830 μm. The 10% MP was enhanced by 8.85%, 5.97%, 5.24%, 4.81%, 4.44%, and 4.34% on the control suspension in the range of <150 μm to >830 μm, respectively. The 15% MP was enhanced by 12.97%, 8.55%, 7.48%, 6.86%, 6.37% and 6.19% on the control suspension in the range of <150 to >830 μm. The 20% MP was enhanced by 17.34%, 11.5%, 10.05%, 9.21%, 8.52% and 8.29% on the control suspension in the range of <150 μm to >830 μm, respectively. 

Since various flocculent structures had different strengths, it was difficult to directly associate the strength of flocculent structures of different sizes with macroscopic flow parameters and microrheological parameters. Figure 11a shows the fitting results of the optimal regression analysis for the fractal dimension and size of the flocculating structure. According to reference [26], the growth in strength of flocculent structures with different sizes conformed to the formula y = bxc, in which the parameter b can be used as the evaluation index of the flocculent structure’s strength. The parameter b in the fitting formula in Figure 11a was proposed and plotted in Figure 11b. The physical meaning of parameter b was to homogenize the flocculent structures of different strengths within the cementitious suspension, that is, the interior structures of the cementitious suspension were composed of flocculent structures of the same strength. The results showed that with the addition of MP replacement content from 5% to 20%, the flocculent structure strength of cementitious suspension increased by 8.8%, 16.8%, 24.8%, and 34.4%, respectively, indicating that the high substitute content of MP had an obvious effect on improving the strength of the flocculent structure. The reason why MP increased the strength of the flocculated structure was that MP enhanced the electrostatic attraction between particles. MP can increase the bonding strength between the cementitious suspension particles, and the particles bond more tightly so that the flocculent structure can resist stronger external stress. In addition, MP with high fineness can also fill the voids of cement particles to enhance the compactness of the structure.

It can be known from the above discussion that parameter a can uniformize the flocculent structure size of the cementitious suspension, and parameter b can uniformize the flocculent structure strength of the cementitious suspension. The bonding strength coefficient was defined as ζ = ab, and the physical meaning was to characterize the flocculent structure’s strength and size inside the cementitious suspension; that is, the interior of the suspension was composed of flocculent structures of the same size and strength. 

**Figure 12:**
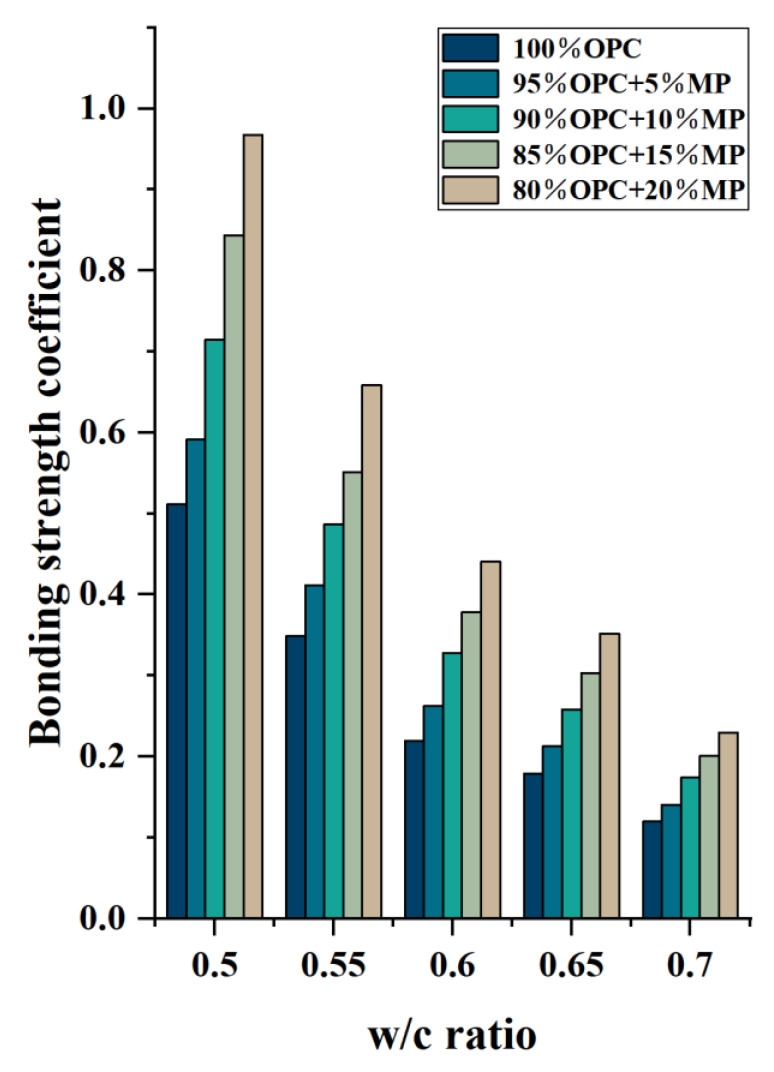
Differentiation of bonding strength coefficient with W/CM at different MP contents.

The bonding strength coefficients are plotted against MP contents for different W/CM ratios in Figure 12. Similar to the above conclusion, the bonding strength coefficient decreased with the increase in W/CM ratio and increased with the increase in MP content. With the increase in MP content from 5% to 20%, the bonding strength coefficients increased by 15.6–19.6%, 39.6–49.2%, 58.2–72.2%, 88.9–100.6% under different W/CM ratios, respectively. High substitute content of MP significantly increased the degree of internal flocculation of the cementitious suspension, while the increase in flocculent structure’s strength and size hindered the flow of the suspension, thereby reducing the macroscopic flow parameters and increasing the microscopic rheological parameters. 

#### 3.2.3. Evaluation of the Relationship between Micro Rheological Parameters, Macro Flow Parameters and Bonding Strength Coefficient and WFT 

**Figure 13:**
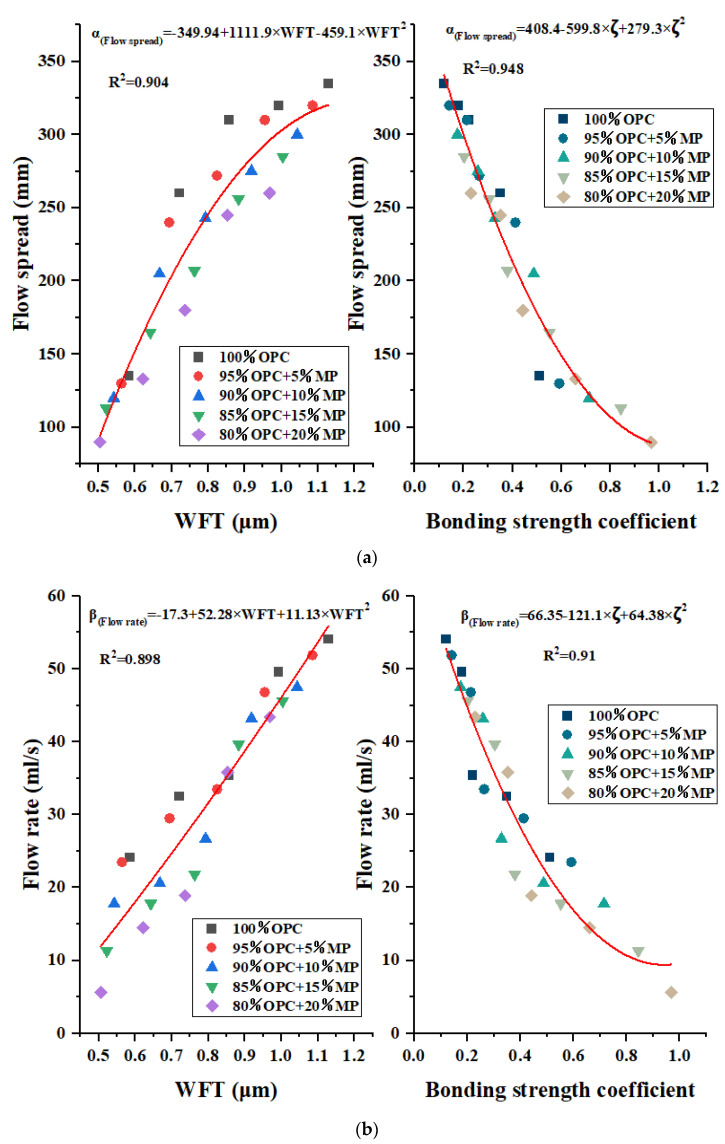
Flow spread and flow rate versus WFT and the bonding strength coefficient. (**a**) Flow spread. (**b**) Flow rate.

Common parameters for evaluating correlation were R, coefficient of determination (R^2^), RMSE, mean absolute error (MAE), relative root mean square error (RRMSE), and relative squared error (RSE) etc., [33]. In this paper, the regression coefficient R^2^ was used to evaluate the relationship between the WFT and bonding strength coefficient and the flow and rheological parameters. The flow rate and flow spread are depicted as a function of WFT and bonding strength coefficient for MP substitutions from 5% to 20% at different W/CM ratios in Figure 13a,b. It can be seen from Figure 13a that the flow spread under different sample codes increased with increasing WFT and decreased with the increase in the bonding strength coefficient. Through regression analysis to fit the best function, the flow spread had a good correlation with WFT and the bonding strength coefficient, R^2^ was 0.904 and 0.948, respectively, and the mathematical model expression was α_(flow spread)_ = −349.94 + 1111.9 × WFT − 459.1 × WFT^2^, α_(flow spread)_ = 408.4 − 559.8 × ζ + 279.3 × ζ^2^. The evolution law of flow spread of cementitious suspension containing MP with bonding strength coefficient and WFT can be well predicted by the mathematical models. The results revealed that both bonding strength coefficient and WFT were the principal factors in determining the static flowability of cementitious suspension. The incorporation of MP affected the flow spread by controlling the particle distance and flocculent structure within the cementitious suspension, and the effect of WFT on flow propagation was weaker than that of the bonding strength coefficient.

Figure 13a indicated that the flow rate under different sample codes increased with increasing WFT and decreased with increasing bonding strength coefficient. Through regression analysis to fit the best function, the flow rate had a good relationship with WFT and bonding strength coefficient, R^2^ was 0.898 and 0.91, respectively, and the mathematical model expression was β_(flow rate)_ = −17.3 + 52.58 × WFT + 11.13 × WFT^2^, β_(flow rate)_ = 66.35 − 121.1 × ζ + 64.38 × ζ^2^. The evolution law of the flow rate of cementitious suspension containing MP with WFT and the bonding strength coefficient can be well predicted by the mathematical models. Similar to static flowability, both bonding strength coefficient and WFT were also the principal factors in determining the dynamic flowability of cementitious suspension. The incorporation of MP affected the flow rate by controlling the particle distance and flocculent structure within the suspension, and the effect of the WFT on the flow rate was slightly weaker than that of the bonding strength coefficient.

**Figure 14:**
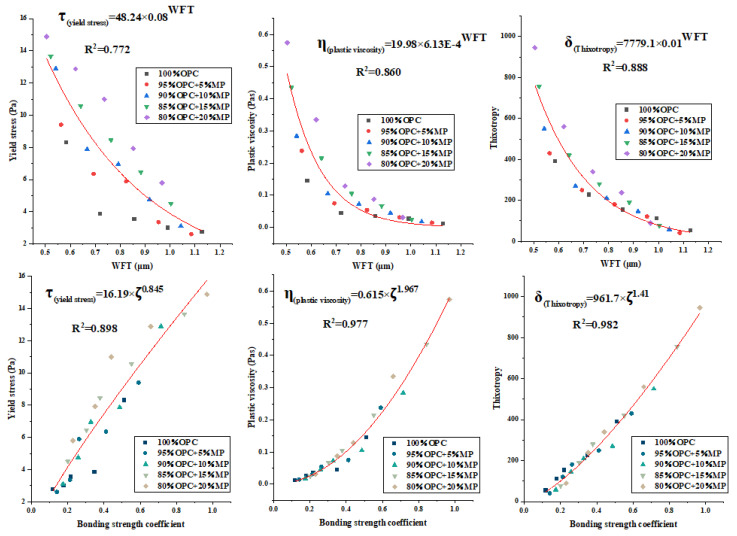
Yield stress, plastic viscosity and thixotropy versus WFT and bonding strength coefficient.

The yield stress, plastic viscosity and thixotropy are depicted as a function of WFT and bonding strength coefficient for MP substitutions from 5% to 20% at different W/CM ratios in Figure 14. The result indicated that the yield stress, plastic viscosity and thixotropy under different sample codes decreased with increasing WFT and increased with an increasing bonding strength coefficient. Under the same WFT, the incorporation of MP can increase the plastic viscosity, yield stress and thixotropy. Under the same bonding strength coefficient, the cementitious suspension containing MP had higher plastic viscosity, higher yield stress and higher thixotropy. The best equations were fitted by regression analysis, and the mathematical models of plastic viscosity, yield stress, and thixotropy and WFT were τ = 45.25 × 0.08^WFT^, η = 19.98 × 6.13× 10^−^^4WFT^, δ = 7779.1 × 0.01^WFT^, respectively, and the regression coefficients were R^2^ = 0.772, R^2^ = 0.860, R^2^ = 0.888, respectively. The mathematical models of plastic viscosity, yield stress, and thixotropy and bonding strength coefficient were τ = 16.19 × ζ^0.845^, η = 0.615 × ζ^1.967^, δ = 961.7 × ζ^1.41^, respectively, and the regression coefficients were R^2^ = 0.898, R^2^ = 0.977, R^2^ = 0.982, respectively. The results revealed that although both the bonding strength coefficient and WFT had a good relationship with thixotropy, plastic viscosity and yield stress, the ability of the bonding strength coefficient to affect rheological parameters was significantly stronger than that of WFT, and the bond strength coefficient regression coefficient R^2^ exceeded the WFT regression coefficient R^2^ by 0.1 or more. Even though both bond strength coefficient and WFT were the main factors that determine the microscopic rheological properties of cementitious suspension, the effect of the bond strength coefficient was stronger than that of WFT, indicating that the change in the flocculent structure within cementitious suspension containing MP was stronger than the change in particle distance. In addition, the high internal specific surface area of MP lead to certain errors in the calculation of WFT. The WFT can reflect the net effect of the filling effect and surface effect of MP, but WFT only considered the external specific area of the particle and did not consider the internal specific surface area, which may lead to an error between the calculated WFT and the real WFT, resulting in a low regression coefficient between WFT and rheological parameters.

### 3.3. Mechanism of MP on Compressive Strength of Hardened Cementitious Suspension

#### 3.3.1. Hydration Activity Contribution Rate

The hydration activity effect ratio strength of admixtures to control paste specific strength can be used as a relative index to evaluate the contribution of the activity effect of auxiliary cementing materials to the compressive strength of control paste. It was defined as the hydration activity contribution rate, and the calculation formula was as follows:

(13) $$P_{a}=\frac{R_{\mathrm{sp}}}{R_{\mathrm{sa}}}=\frac{R_{\mathrm{sa}}-R_{\mathrm{sc}}}{R_{\mathrm{sa}}}$$

(14) $$R_{\mathrm{sa}}=\frac{R_{a}}{q_{0}}$$

(15) $$R_{\mathrm{sc}}=\frac{R_{c}}{100}$$

where *R*_a_ is the absolute strength of hardened slurry containing MP; *q*_0_ is the mass fraction of cement in the gelling system containing MP. *R*_sa_ is the specific strength of hardened slurry containing MP. *R*_c_ is the absolute strength of the reference hardened slurry. *R_sc_* is the specific strength of hardened slurry containing MP.

**Figure 15:**
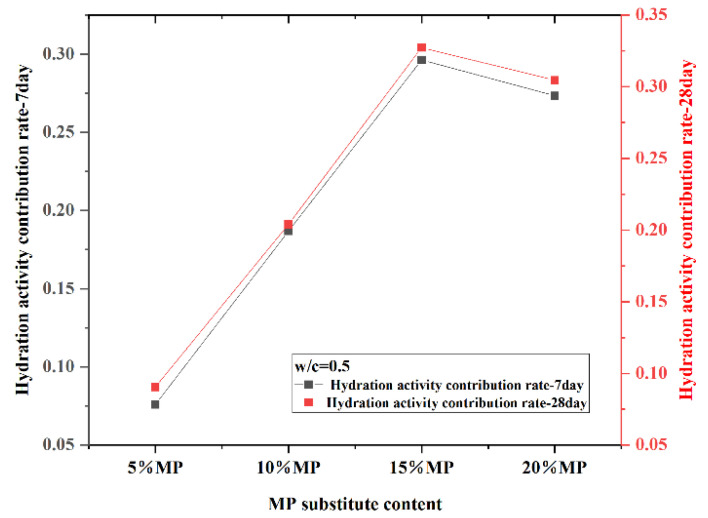
Differentiation of 7-day and 28-day hydration activity contribution rate at different MP contents.

Since the effect of MP on the early and late compressive strength of hardened slurry was the same under different W/CM ratios, only the hydration activity contribution rate of MP was calculated at a W/CM ratio equal to 0.5. The 7-day and 28-day hydration activity contribution rates are plotted against MP contents at a W/CM ratio equal to 0.5 in Figure 15. The results showed that the hydration activity contribution rates of 5%, 10%, 15% and 20% MP at 7 and 28 days were 7.59%, 18.68%, 29.62%, 27.34% and 9.05%, 19.4%, 31.73% and 30.46%, respectively. The higher the hydration activity contribution rate P_a_ value was, the greater the ability of MP to improve the compressive strength of the hardened slurry. The hydration activity contribution rate of MP was consistent with the change rule of 7 and 28-day compressive strength, and the hydration activity contribution rate of MP increased first and then decreased with the increase in MP substitute content from 0–15% and 15–20%. The 15% MP had the highest compressive strength, which indicated that this content of MP had the highest filling effect and the ability to promote hydration. Below the critical value of 15%, MP can effectively reduce structural voids and improve internal paste density degree. When MP was above the critical value of 15%, the ability of MP to increase cement paste strength was less than the negative effect of cement deficiency, so the compressive strength of hardened cement paste decreased. The hydration activity contribution rate of the 28-day MP was larger than that of the 7-day MP due to the volcanic ash effect of MP. The abundant silicon dioxide and aluminum oxide in MP can react with calcium hydroxide to produce calcium silicate hydrate and calcium aluminate hydrate. The formation of hydration products increased the density of the hardened slurry structure and improved the compressive strength.

#### 3.3.2. Mass Water Absorption W, Pore Uniformity α and Average Pore Diameter λ

**Figure 16:**
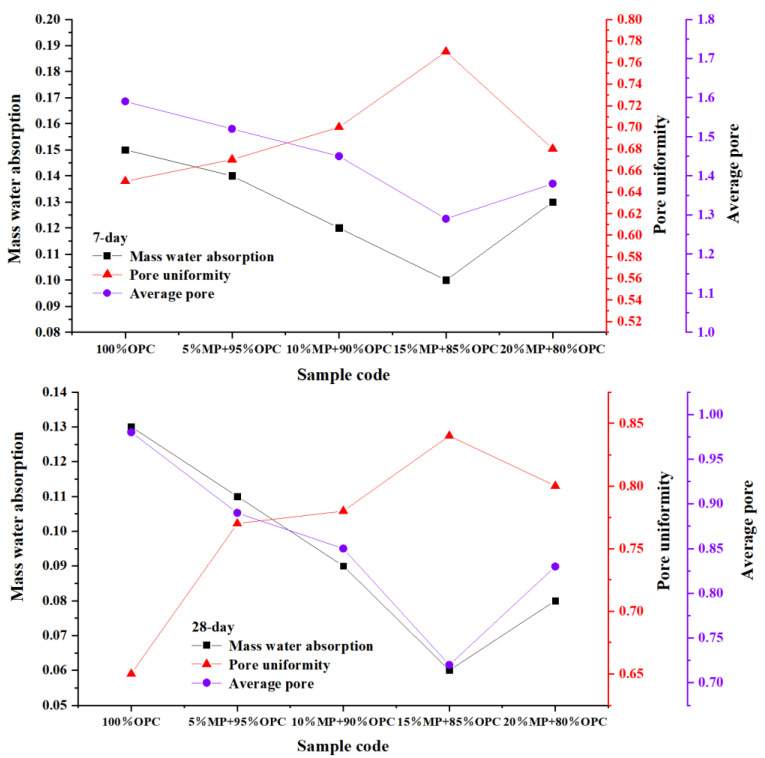
Differentiation of 7-day and 28-day mass water absorption W, pore uniformity α and average pore diameter λ at different MP contents.

The variation of 7-day and 28-day mass water absorption W, pore uniformity α and average pore diameter λ at different MP contents is shown in Figure 16. The mass water absorption rate can reflect the internal voids of the hardened cement paste structure. The lower the mass water absorption rate, the denser the structure of the hardened slurry and the smaller the porosity. The results showed that the replacement of MP can effectively reduce the mass water absorption of the cement paste. The mass water absorption rate of 5%, 10%, 15%, and 20% MP decreased by 6.67%, 20.1%, 33.3%, 30.3% and 15.4%, 30.7%, 53.8%, 38.5%, respectively, compared to the control cement paste at 7 days and 28 days. This was consistent with the compressive strength and hydration activity contribution rate results, where the mass water absorption first decreased and then increased with the increase in MP replacement content from 0 to 15% and from 15% to 20%. The 15%MP had the best ability to reduce mass water absorption; in other words, 15%MP had the best ability to reduce voids of hardened cement paste. In addition, the improvement effect of MP on the mass water absorption of the sample at 28 days is better than that at 7 days, which was attributed to the pozzolanic reaction of MP. The abundant silica and alumina in the MP can react with calcium hydroxide to generate hydration products to enhance the compactness of the internal structure of the hardened paste.

The pore uniformity α and average pore diameter λ were also effective parameters for evaluating the internal void structure of cement paste, where α reflected the uniformity of the pores of the paste, and λ reflected the quantification of the internal pore size of the paste. Both the increase in the pore uniformity α and the decrease in the average pore diameter λ can enhance the internal structure of the slurry and improve the mechanical properties. The average pore diameter λ of 5%, 10%, 15%, and 20% MP decreased by 4.4%, 8.8%, 18.8%, 13.2% and 9.18%, 13.2%, 26.5%, 15.3%, respectively, compared to the control cement paste at 7 days and 28 days, while the average pore uniformity α of 5%, 10%, 15%, and 20% MP increased by 3.1%, 7.7%, 18.4%, 8.6% and 18.4%, 20.1%, 29.3%, 23.07%, respectively, compared to the control cement paste at 7 days and 28 days. The results showed that the variation law of pore uniformity α and average pore diameter λ with MP was completely consistent with the compressive strength, hydration activity contribution rate, and water absorption rate. The internal structure of the paste > 50 nm can be called harmful pores, and the ability of MP to reduce the pore size and improve the uniformity of the pores was beneficial to improving the mechanical properties, which explains the mechanism of MP on the compressive strength, and 15% MP was also the optimal ratio that can reduce pore size and improve pore uniformity. The effect of MP on improving pore size and pore uniformity is better at 28 days than at 7 days.

In conclusion, MP had the ability to enhance the compressive strength of hardened slurry, which was attributed to the pozzolanic effect of MP, thereby improving the paste porosity, pore size and pore uniformity and enhancing the compactness of the structure. The improvement effect of MP was the best at the substitution of 15% and enhanced with the increase in curing age.

## 4. Conclusions

From the above discussion, the following conclusions can be obtained:

1. The addition of MP can reduce the macro flow parameters of cementitious suspension and increase the micro rheological parameters. As MP replacement increased from 5% to 20%, the flow spread decreased in the range of 2.4–48.8%, the flow rate decreased in the range of 2.1–76.7%, the yield stress increased in the range of 5.1–234.2%, the plastic viscosity increased in the range of 1.2–661.3%, the thixotropy increased in the range of 1.8–146.69%, and the compressive strength increased in the range of 2.8–31.5%.
2. With the addition of MP from 5% to 20%, the packing density increased by 0.75%, 1.5%, 2.2%, and 3.2%, while the void ratio decreased by 1.6%, 3.2%, 4.7%, and 6.6%. Considering the net effect of the filling effect and surface effect, the addition of MP reduces the WFT of the particles, which explained the mechanism of MP affecting microscopic rheological parameters and macroscopic flow parameters from the particle distance.
3. The addition of MP increased the flocculent structure’s size and strength, and this effect was enhanced with the increase in MP replacement. The incorporation of MP decreased the flocculent structure in the <150 μm range and increased the flocculent structure in the >830 μm range at various W/CM ratios. The mechanism of MP affecting microscopic rheological parameters and macroscopic flow parameters was explained from the flocculent structure.
4. The addition of MP reduced the average pore diameter λ and mass water absorption W and increased pore uniformity α and the hydration activity contribution rate, which explained the improvement of MP’s compressive strength from the perspective of water absorption kinetics and hydration. Among them, 15% MP had the best mechanical and microscopic improvement effect.
5. The WFT and bonding strength coefficient can effectively characterize flow parameters and rheological parameters, but only mathematical models were established. Based on this evaluation system, a rheological theoretical physical model can be established based on further studies on hydration kinetics, collision mechanics and kinetics. This part of the content is currently in the blank stage of the research field and can be completed in the future.

## Figures and Tables

**Table 1:** Chemical compositions of MP and OPC.

Phase	Mass Percentage (%)	
	OPC	MP
SiO_2_	13.9	43.68
Al_2_O_3_	3.05	44.96
Fe_2_O_3_	4.80	1.58
CaO	72.37	0.33
MgO	1.07	4.26
Na_2_O	0.16	<0.01
SO_3_	2.63	2.64
K_2_O	0.97	0.45
P_2_O_5_	0.23	<0.01
TiO_2_	0.35	1.66
MnO	0.30	0.04
ZrO_2_	0.02	<0.01
SrO	0.10	<0.01
Cl	0.02	<0.01

**Table 2:** Variation of flow rate and flow spread with W/CM at various MP contents.

Sample Code	w/c Ratio	Flow Spread (mm)	Flow Rate (mL/s)
100% OPC	0.5	135	24.1
95% OPC + 5% MP	0.5	130	23.5
90% OPC + 10% MP	0.5	120	17.8
85% OPC + 15% MP	0.5	113	11.3
80% OPC + 20% MP	0.5	90	5.6
100% OPC	0.55	260	32.5
95% OPC + 5% MP	0.55	240	29.5
90% OPC + 10% MP	0.55	205	20.6
85% OPC + 15% MP	0.55	165	17.8
80% OPC + 20% MP	0.55	133	14.5
100% OPC	0.6	310	35.39
95% OPC + 5% MP	0.6	272	33.5
90% OPC + 10% MP	0.6	243	26.7
85% OPC + 15% MP	0.6	207	21.8
80% OPC + 20% MP	0.6	180	18.9
100% OPC	0.65	320	49.56
95% OPC + 5% MP	0.65	310	46.8
90% OPC + 10% MP	0.65	275	43.2
85% OPC + 15% MP	0.65	256	39.65
80% OPC + 20% MP	0.65	245	35.84
100% OPC	0.7	335	54.1
95% OPC + 5% MP	0.7	320	51.89
90% OPC + 10% MP	0.7	300	47.5
85% OPC + 15% MP	0.7	285	45.6
80% OPC + 20% MP	0.7	260	43.4

**Table 3:** Variation of 7-day and 28-day compressive strength with W/CM at various MP contents.

Sample Code	w/c Ratio	7-Day Compressive Strength (MPa)	28-Day Compressive Strength (MPa)
100% OPC	0.5	17.8	27.38
5% MP + 95% OPC	0.5	18.3	28.6
10% MP + 90% OPC	0.5	19.7	29.7
15% MP + 85% OPC	0.5	21.5	33.6
20% MP + 80% OPC	0.5	19.6	31.5
100% OPC	0.55	14.2	21.38
5% MP + 95% OPC	0.55	15.1	23.5
10% MP + 90% OPC	0.55	16.2	25.4
15% MP + 85% OPC	0.55	17.6	27.6
20% MP + 80% OPC	0.55	17.1	26.8
100% OPC	0.6	10.3	16.15
5% MP + 95% OPC	0.6	10.8	17.8
10% MP + 90% OPC	0.6	11.6	18.9
15% MP + 85% OPC	0.6	13.2	20.4
20% MP + 80% OPC	0.6	11.9	19.6
100% OPC	0.65	8.1	13.5
5% MP + 95% OPC	0.65	8.7	14.5
10% MP + 90% OPC	0.65	9.2	15.8
15% MP + 85% OPC	0.65	10.65	17.1
20% MP + 80% OPC	0.65	10.1	16.2
100% OPC	0.7	7.5	10.734
5% MP + 95% OPC	0.7	7.8	11.2
10% MP + 90% OPC	0.7	8.3	12.7
15% MP + 85% OPC	0.7	8.9	13.8
20% MP + 80% OPC	0.7	7.8	13.2

**Table 4:** Variation of flocculation structure mass fraction with W/CM at different MP contents.

w/c Ratio	Sample Code	Flocculation Structure Mass Fraction				
		Flocculation Structure Size (μm)				
		150 μm	300 μm	425 μm	600 μm	830 μm
	C-0-0	0.805	0.545	0.245	0.108	0.057
	C-5-0	0.835	0.576	0.259	0.123	0.065
0.5	C-10-0	0.889	0.583	0.323	0.169	0.089
	C-15-0	0.921	0.635	0.413	0.176	0.095
	C-20-0	0.932	0.678	0.459	0.189	0.105
	--	150 μm	300 μm	425 μm	600 μm	830 μm
	C-0-0	0.754	0.316	0.097	0.083	0.046
	C-5-0	0.797	0.343	0.115	0.092	0.048
0.55	C-10-0	0.823	0.368	0.136	0.112	0.069
	C-15-0	0.851	0.389	0.143	0.189	0.073
	C-20-0	0.875	0.419	0.154	0.125	0.079
		150 μm	300 μm	425 μm	600 μm	830 μm
	C-0-0	0.669	0.125	0.065	0.031	0.024
	C-5-0	0.723	0.132	0.071	0.042	0.028
0.6	C-10-0	0.751	0.157	0.105	0.059	0.039
	C-15-0	0.765	0.189	0.115	0.068	0.04
	C-20-0	0.786	0.225	0.126	0.075	0.041
	--	150 μm	300 μm	425 μm	600 μm	830 μm
	C-0-0	0.57	0.096	0.048	0.026	0.015
	C-5-0	0.598	0.105	0.057	0.032	0.02
0.65	C-10-0	0.628	0.113	0.069	0.054	0.024
	C-15-0	0.654	0.125	0.078	0.068	0.031
	C-20-0	0.675	0.138	0.097	0.072	0.034
	--	150 μm	300 μm	425 μm	600 μm	830 μm
	C-0-0	0.394	0.051	0.0347	0.0187	0.012
	C-5-0	0.405	0.057	0.046	0.019	0.015
0.7	C-10-0	0.435	0.079	0.057	0.021	0.019
	C-15-0	0.454	0.083	0.069	0.025	0.02
	C-20-0	0.476	0.086	0.076	0.028	0.021

## Data Availability

Not applicable.
